# An Inexpensive Cardiovascular Flow Simulator for Cardiac Catheterization Procedure Using a Pulmonary Artery Catheter

**DOI:** 10.3389/fmedt.2021.764007

**Published:** 2021-10-28

**Authors:** Annika Johnson, Grace Cupp, Nicholas Armour, Kyle Warren, Christopher Stone, Davin Lee, Nicholas Gilbert, Chris Hammond, John Moore, Youngbok (Abraham) Kang

**Affiliations:** ^1^Department of Mechanical, Civil, and Biomedical Engineering, George Fox University, Newberg, OR, United States; ^2^TZ Medical Inc, Portland, OR, United States

**Keywords:** cardiac catheterization, catheter, femoral and radial access, vascular flow simulator, cardiovascular

## Abstract

Cardiac catheterization associated with central vein cannulation can involve potential thrombotic and infectious complications due to multiple cannulation trials or improper placement. To minimize the risks, medical simulators are used for training. Simulators are also employed to test medical devices such as catheters before performing animal tests because they are more cost-effective and still reveal necessary improvements. However, commercial simulators are expensive, simplified for their purpose, and provide limited access sites. Inexpensive and anatomical cardiovascular simulators with central venous access for cannulation are sparse. Here, we developed an anatomically and physiologically accurate cardiovascular flow simulator to help train medical professionals and test medical devices. Our simulator includes an anatomical right atrium/ventricle, femoral and radial access sites, and considers the variability of arm position. It simulates physiological pulsatile blood flow with a setting for constant flow from 3 to 6 L/min and mimics physiological temperature (37°C). We demonstrated simulation by inserting a catheter into the system at radial/femoral access sites, passing it through the vasculature, and advancing it into the heart. We expect that our simulator can be used as an educational tool for cardiac catheterization as well as a testing tool that will allow for design iteration before moving to animal trials.

## Introduction

**Figure 1:**
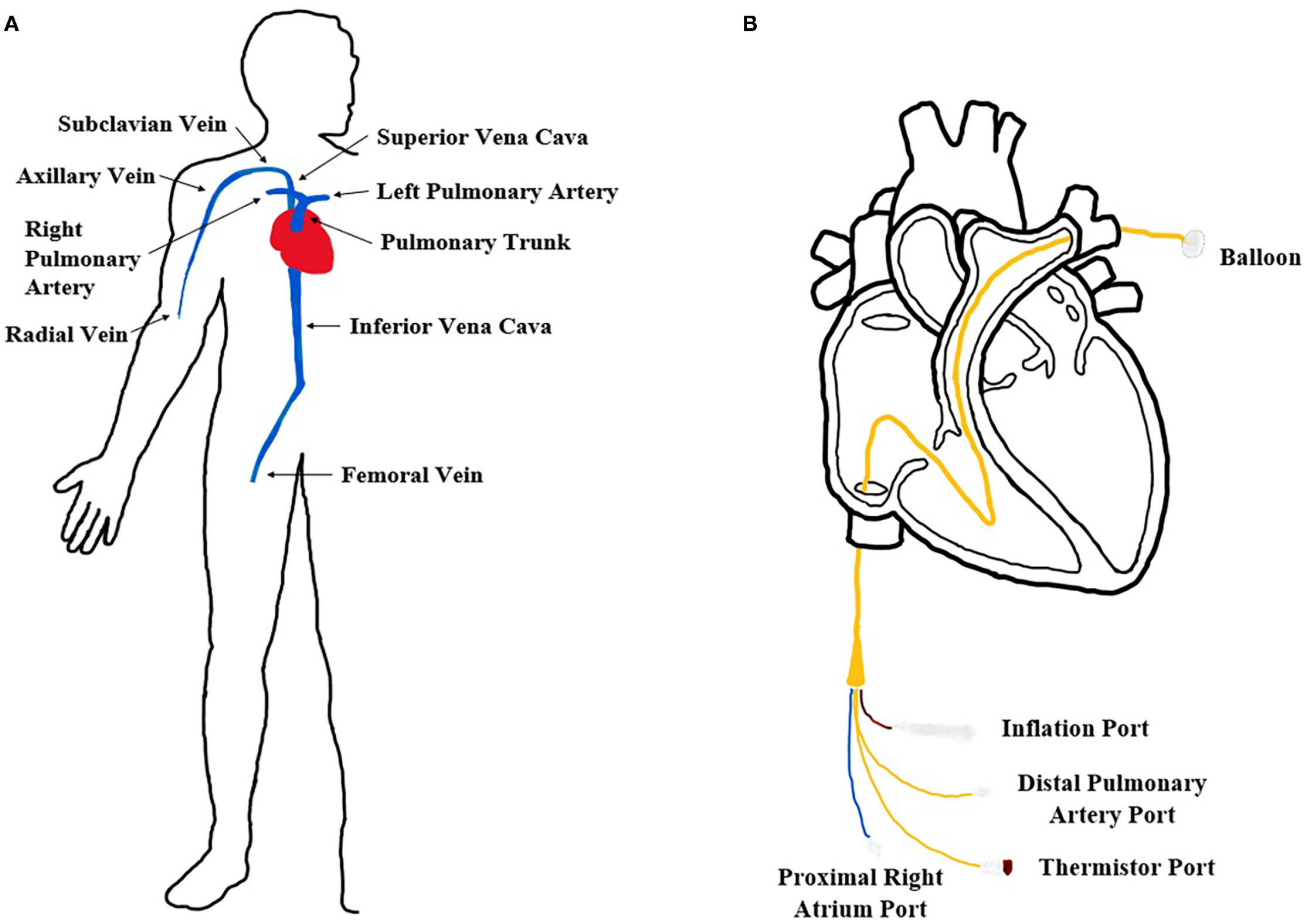
Cardiovascular system and catheterization procedure. **(A)** Diagram of the human *in vivo* cardiovascular system in our body. **(B)** Expanded femoral insertion pulmonary artery catheterization procedure diagram.

Cardiac catheterization associated with heart disease treatment such as coronary angioplasty and coronary stenting has been conducted more than 1,000,000 times every year in the United States (1, 2). It is an interventional catheterization procedure which avoids surgically opening the chest and allows for the insertion of a catheter into an artery or vein that is then guided to the heart (Figure 1) (3, 4). This procedure is commonly used to diagnose cardiovascular conditions, implant certain medical devices, repair heart defects, and take samples of blood or heart muscles. Although the catheterization procedure is effective and essential in cardiac disease treatment, procedure failure and tissue damage from multiple cannulation trials and complications including hemorrhage and thrombosis have often occurred (5). To minimize these risks and incidents, doctors and other medical professionals need to be adequately trained before performing the procedure on patients. To meet this need, medical cardiovascular flow simulators and virtual reality simulators are widely used. Further, medical simulators are essential for testing medical devices in case of unavailable animal models. Another benefit of these tools includes decreasing development expenses for devices that require FDA approval. As a result, some medical simulators have been developed and are available on the market.

About two decades ago, Anderson et al. presented a working prototype of an augmented reality based vascular catheterization simulator using computational modeling of human anatomical images (6). A more advanced virtual reality simulator modeled the behavior of a virtual catheter responding to instrument manipulations in the simulated cardiovascular flow system. This simulator provided a virtual three-dimensional (3-D) environment to trace surgeons' consoled vessel trajectories and to mimic the elasticity of the vessel wall *via* a haptic force producer (7, 8). Along with the virtual simulator, some studies have focused on a computational simulation of blood flow dynamics in a cardiovascular system (9–12). Predicting the fluid dynamics including flow distribution, flow rate, and the location of turbulence was found beneficial during the insertion of a curved arterial cannula and cardiopulmonary bypass in the aortic arch. However, virtual simulators and computational simulations are limited because they do not provide the physical tools necessary for mastering the hands-on technique of cardiac catheterization.

Non-virtual reality simulation models have been developed for several decades and widely used for more practical training in clinical procedure and design verification of medical devices. Simplified vascular flow simulators without the heart model were developed by Eason et al. (13, 14). These models replicate central venous and arterial pulsatile blood flow patterns through a femoral access point and change pulse intensity with cannulation. Rebecca et al. presented the arterial line insertion simulator that generated pulsatile flow and allowed radial access at the arm for catheter insertion performed with a guidewire (15). Unlike the simplified vascular flow simulators, Rotman et al. demonstrated a more accurate human cardiovascular flow simulator that calcified aortic valve models were incorporated into (16). It is a realistic vascular replicator to test performance of transcatheter aortic valve replacement (TAVR). More recently, Zhu et al. presented an *ex-vivo* aortic simulator to mimic human aortic regurgitation from cusp prolapse using porcine aortic valves for evaluation of valve biomechanics and surgical repair techniques (17). However, these were specialized and designed for valve replacement/repair, not catheterization procedures because blood vessels were simplified or omitted.

Several cardiovascular flow simulators are commercially available on the market. The Replicator PRO in Mentice Inc. is an advanced endovascular physiological flow system for replicating an anatomical four chamber heart with heart valves, silicone vasculature lines, and multiple access points (16, 18, 19). The ViVitro endovascular simulator in ViVitro Labs Inc. is a configurable vascular vessel system based on a modular platform (17, 20). The physiological vascular vessels and pulsatile flow patterns are re-configurable according to intention of use and catheter position and procedure deployment can be tracked using a digital camera. The Mock Circulatory Loop in BDC Laboratories Inc. provides a physiological function to control pressure, temperature, and pulsatile flow conditions of the human circulatory system (21). However, these systems excluded or simplified the heart element, or minimized the number of access points to narrow down the variables that would be affected during surgical procedure simulation. While these systems provide a tracking tool for the device such as an X-ray, it is expensive and hard to visually inspect a device's movement (e.g., catheter) through vessels due to its non-transparency.

Thus, there is still a need to develop an anatomically accurate cardiovascular flow simulator with physiological fluidic function and multiple access points for cardiac catheterization that can be used for testing medical devices (e.g., catheter) and training clinicians (e.g., catheterization procedure at vein vessel).

In our study, we focused on developing a cardiovascular flow simulator for catheterization procedures using a pulmonary artery (PA) catheter (Figure 1). A long, thin, flexible PA catheter is an invasive medical device designed to measure vital human parameters (blood pressure, oxygenation, etc.) for patients in critical care. Specifically, flow-directed PA catheters are typically inserted in either the radial or femoral vein and hand-guided through the venous system until the opening of the right atrium. A balloon at the tip of the PA catheter is then inflated and the flow of the heart guides the catheter to a pulmonary artery where measurements are obtained. Since the device is invasive, multiple successful animal (e.g., pig) tests must be performed before FDA approval which can be costly for medical companies. Thus, a cardiovascular flow simulator is commonly used before animal testing to evaluate the behavior and performance of a PA catheter as it passes through the chambers of the right side of the heart from a femoral or radial access site.

**Figure 2:**
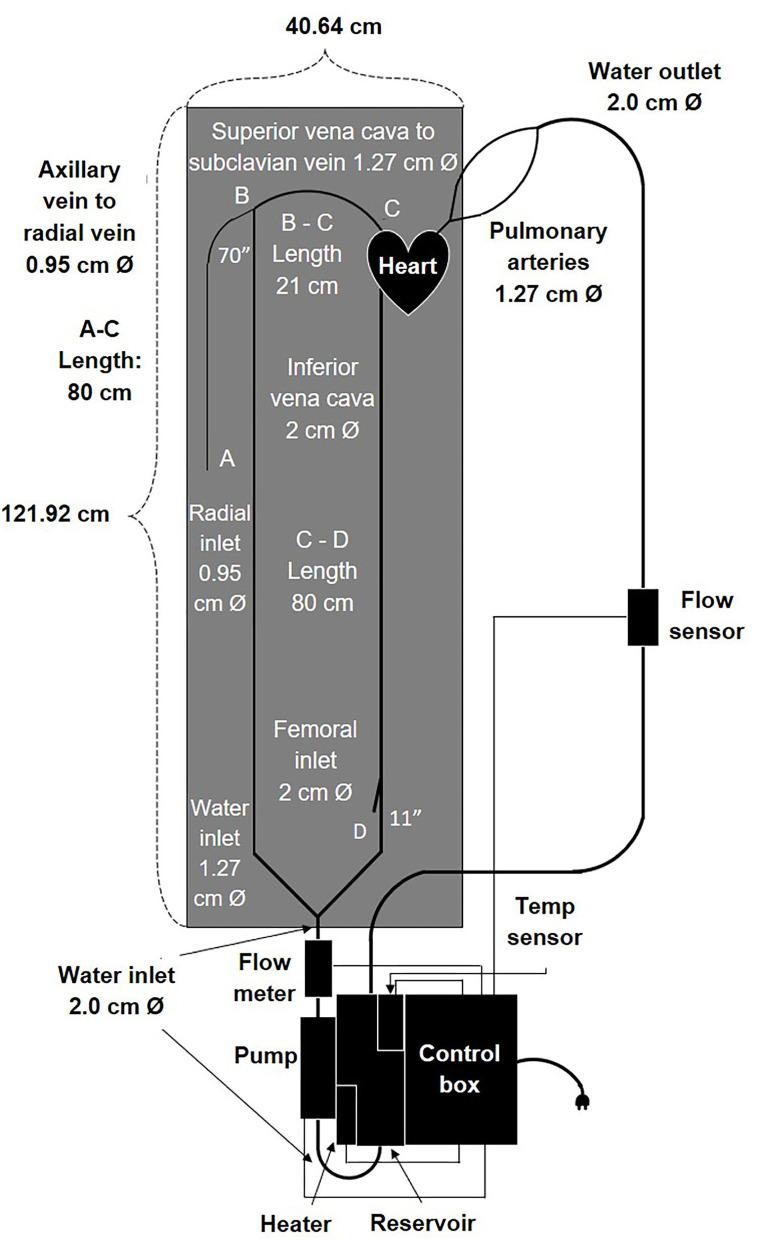
Schematic drawing including components and dimensions of the cardiovascular flow simulator.

With this necessity, we designed the cardiovascular flow simulator that includes the right side of the heart with cardiovascular lines to mimic the blood circulatory system *in vivo* (Figure 2). We reconstructed an anatomical 3D heart model by analyzing the human heart geometries from MRI images and assembled it with all the mechanical parts including vinyl tubing, a pump, and circuits for the various sensors and controlled components. We then tested physiological pulsatile flow at body temperature and simulated catheter insertion through femoral and radial access sites in the system.

In summary, we successfully developed an anatomical cardiovascular flow simulator that reflected human hemodynamics and evaluated the performance of a flow-directed catheter in the right chambers of the heart from both femoral and radial access sites. We expect that our model would be used for evaluating the performance of different flow-directed catheter designs in a laboratory environment. Additionally, we anticipate that it will be a medical simulation tool for catheterization procedure training during routine practice and when clinically relevant animal models, the *in vivo* model, and the *ex-vivo* models are not available.

## Materials and Methods

### Design Specification

We designed a cardiovascular flow simulator by evaluating the cardiovascular catheterization procedure which involves inserting a PA catheter into either a radial or femoral vein and then guiding it through the right atrium and right ventricle of the heart to a pulmonary artery (Figure 2). The heart design in our vascular flow simulator focused on only the right-side chambers of the heart. Our simulator was designed to meet specific requirements including: anatomical accuracy, physiological flow patterns at body temperature, inexpensive reproducibility, and easy drainage, maintenance, and transportation. In addition, our device provides access sites at the femoral and radial locations and enables a user to observe catheter motion in the vessels and to inspect defects. Moreover, each component of the product can withstand the impact force exerted by the object when it falls from a reasonable height. The detailed specifications are listed in Table 1.

**Table 1:** Design specification and criteria for the cardiovascular flow simulator.

**No**	**Item**	**Design specification**	**Design criteria**
1	Anatomical model	The device needs to replicate proper flow dynamics and physiology of an anatomically accurate adult human heart.	As anatomically correct as possible heart and vasculature.
2	Maintenance	The device needs to be easy to maintain.	Repair and replacement.
3	Visualization	The material has to meet specific characteristics to observe the catheter in the working device.	Transparent material
4	Safety	The device needs to follow proper safety regulations. The GFCI and built-in fusing must protect the user and the system from harm.	Meet safety guidelines (22–26)
5	Durability	The device must be able to withstand a fall from a reasonable height and many operating hours.	Drop test from about 1 m height.
6	Operating temperature	The operating temperature of the system must be about 37°C.	37 ± 1°C
7	Friction	The material must have a friction coefficient close to the friction coefficient of internal anatomical vasculature.	0 < Friction coefficient < 0.5 (27)
8	Flow	The pump must function at the desired flow rates for both pulsatile and continuous flow. The flow must run through the system with minimal-to-no bubbles and leaking.	3–6 L/min with pulsatile pattern Removable bubbles and no leaking

### Heart Design and Fabrication

**Figure 3:**
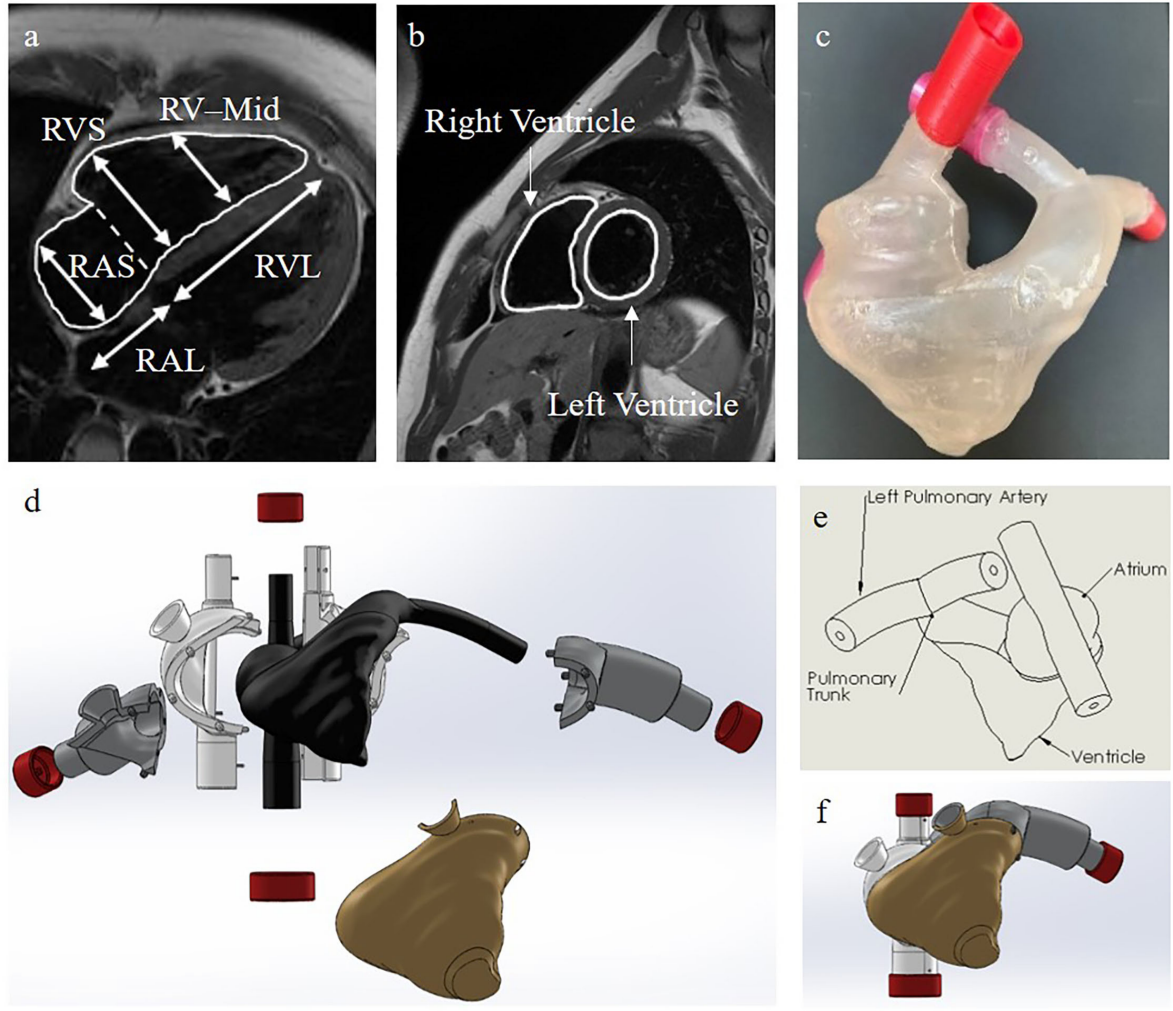
Our heart model. **(a)** Side view of the internal right side of the heart with the dimensions used for verification of our heart model in Table 2. RVS, Right Ventricular Short Axis; RAS, Right Atrial Short Axis; RV-Mid, Right Ventricular Midway Axis; RVL, Right Ventricular Long Axis; RAL, Right Atrial Long Axis (34). **(b)** Cross-sectional MRI images of the heart for 3D reconstruction (34). **(c)** Our final casted heart model. **(d)** A color-coded exploded view of the internal and external SolidWorks models of the molds used for casting our final heart model. Black is for the internal heart mold, gold is for the ventricle mold, gray is for the pulmonary artery molds, white is for the atria and vena cava molds, and red is for the end caps. **(e)** A labelled schematic drawing of the SolidWorks reconstructed internal heart mold. **(f)** The collapsed view of the SolidWorks molds.

To design an anatomically accurate heart model, we initially found basic dimensions of the internal anatomy of the heart measured from silicone molds of cadaver hearts (Table 2, Figure 3) (28–33). Although such dimensions were helpful for verifying already reconstructed hearts, they didn't provide enough information about the shape of the right ventricle, atrium, pulmonary trunk, and vena cava to reconstruct the heart in 3D. To get more information, we accessed the AMRG Cardiac Atlas which is an open source set of MRI images of a healthy 25 year-old male's heart (34). Using MATLAB, we analyzed the many MRI scans and selected a set of 20 cross-sectional images where the various heart structures were easily identifiable (Figure 3). Further, from MATLAB we extracted the dicom voxel size and patient orientation for 3D reconstruction of the heart. We then imported the MRI scans into Solidworks at the correct orientation and vertical spacing. Using the spline and loft tools we reconstructed the entire internal right-side geometry of the heart, the pulmonary trunk, the pulmonary arteries, and the superior & inferior vena cava (SVC & IVC) (Supplementary Figure 1). In order to obtain a hollow, visually-clear, watertight model of the heart, with a physiological frictional coefficient, we chose to cast our final heart mold out of Smooth-On's Crystal Clear 2,000 Polyurethane (Smooth-On, PA). In Solidworks, we created an external mold with a 5 mm gap for the walls of the final heart. This mold was broken into pieces, with pouring funnels for casting around the internal mold (Figure 3). The internal and external molds were then 3D-printed with a water-soluble filament called Prima Select PVA + Soluble Support (Prusa Research, Czech Republic) using a 3D printer (Prusa Research, Czech Republic). We then used water to smooth the internal surface of the heart and minimize the coefficient of friction. To ensure the mold won't adhere to the final heart model, three coats of wax and one coat of release spray was used on the mold prior to pouring the polyurethane. We then assembled the mold and sealed the seams with silicone and tape. The polyurethane was mixed at a ratio of 100A:90B at 73°F slowly for 3 min according to the manufacturer's instruction. To remove bubbles, we put the polyurethane mixture in a vacuum chamber for 5 min and then poured the polyurethane into the mold and allowed it to cure for at least 24 h. Dissolving the water-soluble mold then required submerging it entirely in water for ~48 h, exchanging the water every few hours. Finally, after the water-soluble material completely dissolved, we obtained a visually clear, 5 mm thick hollow cast of the internal anatomy of the right side of the heart.

**Table 2:** The dimension of the heart model.

**Dimension label**	**Smaller heart**	**Larger heart**	**Average heart**	**Our heart**
Right atrial long axis	4.43 ± 0.40 cm	4.65 ± 0.33 cm	4.51 ± 0.47 cm	4.45 cm
Right atrial short axis	4.63 ± 0.31 cm	4.96 ± 0.29 cm	4.79 ± 0.47 cm	5.08 cm
Right atrial area	18.72 ± 1.60 cm^2^	19.97 ± 1.97 cm^2^	19.53 ± 2.41 cm^2^	-
Right ventricular long axis	7.55 + 0.46 cm	8.58 ± 0.40 cm	8.04 ± 0.94 cm	7.62 cm
Right ventricular short axis (Maximal)	4.53 ± 0.35 cm	4.73 ± 0.33 cm	4.62 ± 0.44 cm	5.00 cm
Right ventricular short axis (Midway)	3.56 ± 0.28 cm	3.77 ± 0.39 cm	3.72 ± 0.44 cm	3.81 cm
Right ventricular area	25.81 ± 2.85 cm^2^	30.57 ± 3.53 cm^2^	28.53 ± 5.57 cm^2^	-

*The dimension of the smaller and larger heart was obtained from measurements of silicone casts of cadaver hearts of various sizes from literature (28). We calculated the average of the smaller and larger heart for a set of more general values to compare with. These measurements were used for further verification of accurate anatomical dimensions on our heart. The values we obtained using callipers on our final manufactured heart are also displayed*.

### Tubing and Connectors for Vascular Flow Line

**Figure 4:**
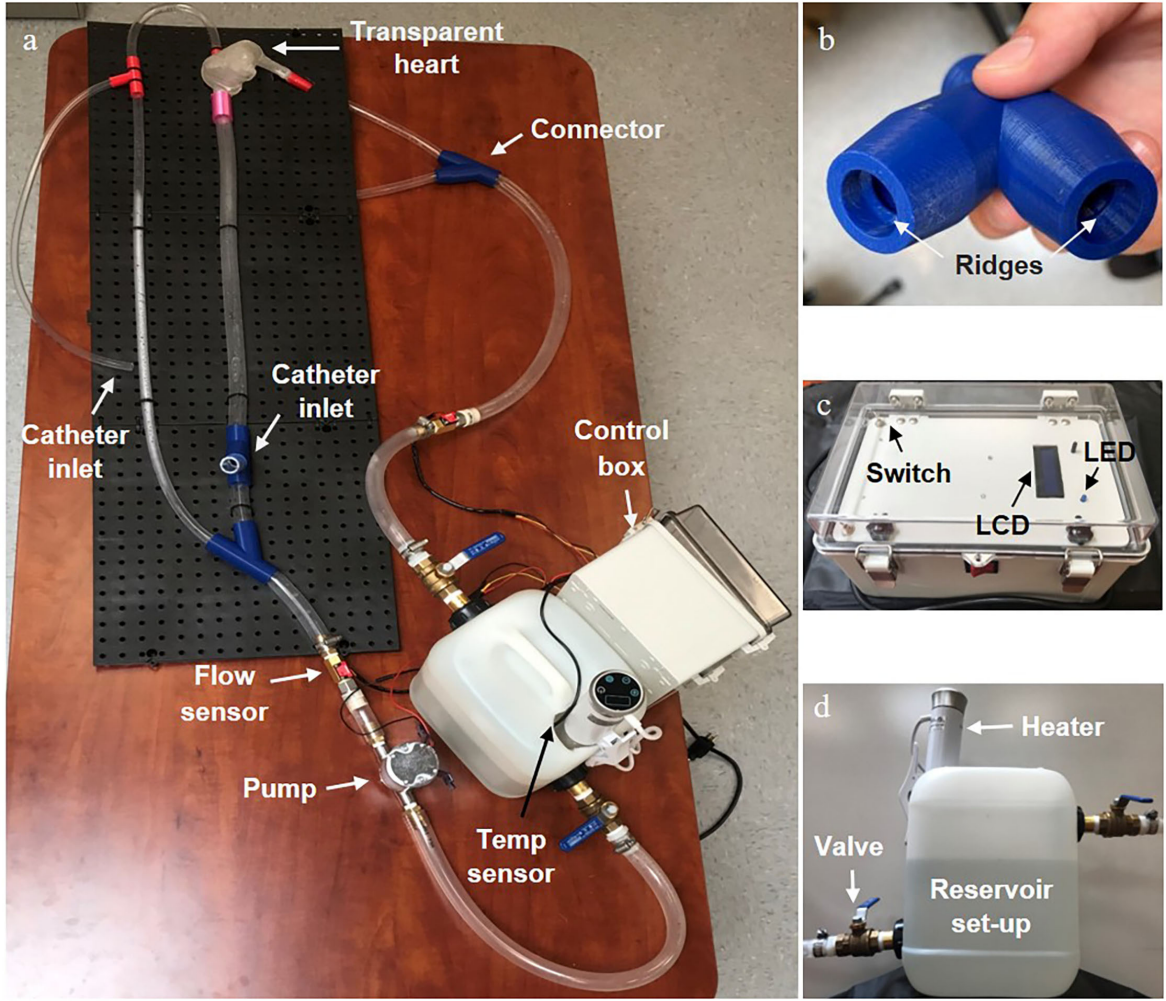
Final physical vascular flow simulator. **(a)** The entire set-up with the main individual components labelled. **(b)** An enlarged view of the 3D printed connector showing the ridges that created a water tight seal with the tubing. **(c)** A front view of the control box showing the LCD display screen, state switches on the opposite side corners, and LED status indicator. **(d)** A side view of the reservoir set up with the heating element and valves clearly visible.

To develop vascular blood vessels, we used vinyl tubing with appropriate anatomical vessel sizes (Table 3) because it provides transparency and low friction loss compared to silicone (35–38). One vinyl tube of ½ inch size connected to the Superior vena cava (SVC) outlet of the 3D heart model. It then connected to a 3/8 inch internal diameter tube for the radial access site and continued to the pump *via* an angle T-connector (Figures 2, 4). Another vinyl tube of ¾ inch size for the femoral vein access site was connected to the Inferior vena cava (IVC) outlet of the 3D heart model. The pulmonary arteries of the heart were each connected to a ½ inch diameter vinyl tube and rejoined into one ½ inch tube *via* a Y-connector before entering the reservoir (Figures 2, 4). Fluid from the reservoir was supplied to each venous flow inlet *via* a bifurcation of SVC and IVC in a loop following the pump. Rubber stoppers are used to block the access sites when not in use.

**Table 3:** The internal diameter dimensions of anatomical vessels and our vascular system.

**Vessel name**	**Anatomical diameter**	**Diameter of our system**
Right femoral vein	1.10 ± 0.10 cm (29)	2.0 cm^*^
Inferior vena cava	1.75 ± 0.25 cm (27)	2.0 cm
Superior vena cava	1.45 ± 0.20 cm (26)	1.27 cm
Right subclavian vein	1.33 ± 0.20 cm (24)	1.27 cm
Right axillary vein	1.04 ± 0.18 cm (31)	0.95 cm
Pulmonary trunk	2.48 ± 0.51 cm (25)	2.54 cm
Right pulmonary artery	1.28 ± 0.28 cm (26)	1.27 cm
Left pulmonary artery	1.22 ± 0.42 cm (26)	1.27 cm

*From the literature review, we found the internal diameter dimensions of anatomical cardiovascular vessels that were measured through cadavers, MRI imaging techniques, ultrasound imaging techniques, etc. These were used for verification of vessel dimensions of our system*.

* *The femoral diameter is inconsequential because it is very short and just for the catheter insertion port (Tuohy-Borst)*.

To connect tubing without creating any irregular flow disturbances and allow a catheter to move easily through the device, we specially designed an external connector with an internal ridge for form-fitting the tubing into the connector (Figure 4). We 3D-printed it with PLA material making sure to increase the infill and wall thickness to make it waterproof.

### Temperature and Flow Components

To monitor flow rate, we installed two Hall-Effect flow sensors (YF-B2, Seeed Technology Co., Ltd), one right after the pump outlet and one immediately before fluid reentered the reservoir. In order to simulate body temperature, we used a Sous Vide heating element (Lovisida, Leyida Industrial Co., China) in a water reservoir of 2.5 gallons (UN-compliant shipping jug, McMaster-CARR) with a compatible digital temperature sensor (DS18B20, Adafruit) to achieve 37°C for the system. The sous vide was connected to a 120-volt DIN terminal strip in the control box which contains all of the power and IC elements. To introduce fluid flow in the loop, an impeller pump (UP-1 Bilge, Marco) was installed next to a fluid reservoir and controlled *via* an Arduino Uno microcontroller (Arduino, MA).

### Flow Test

**Figure 5:**
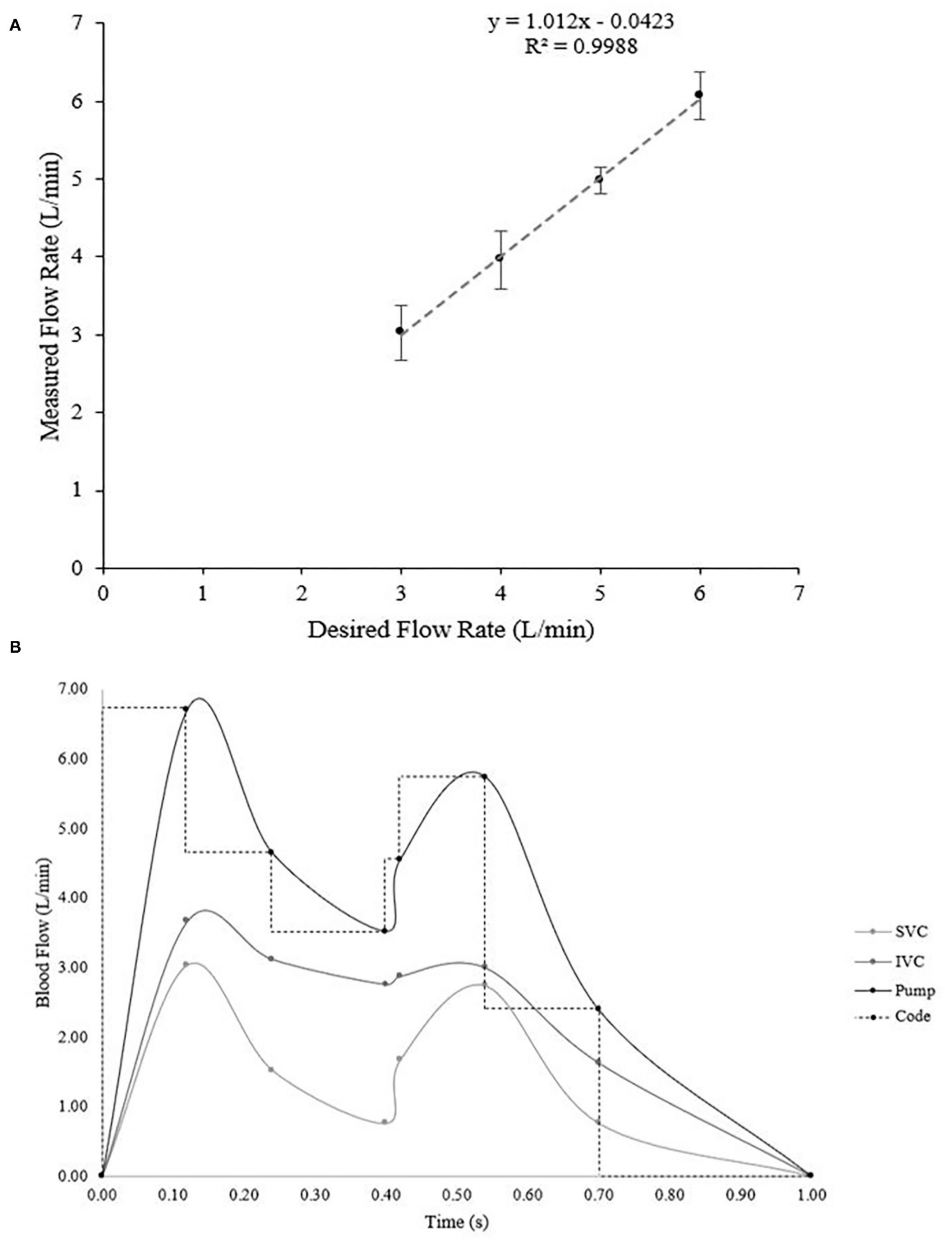
**(A)** Verification of the flow rate during various constant flow rate trials ranging from 3–6 L/min. Ideally, the linear relationship would be y = x, which our calculated equation of y = 1.012 × −0.0423 is very close to (R-squared value in linear regression is 0.9988). **(B)** Calculated flow profiles during pulsatile flow. The inferior and superior vena cava profiles were obtained based on data from literature (39, 40) and then the pump output necessary to create those two profiles was calculated *via* fluid dynamics principles. Then the Arduino code was set to mimic the function in steps.

Once we assembled all the parts and filled the reservoir, the simulator was suffused with water by the pump. Air bubbles trapped in the loop were removed by lifting and lowering the device as needed to allow the bubbles to flow out of the system. Before the flow test, we estimated the correction factor needed to be used in the code to ensure the desired input was what the pump actually output. We controlled the pump using an Arduino Uno microcontroller which operated a transistor (e.g., MOSFET) that caused various pulse width modulation (PWM) cycles to create different flow rates. The code was programmed to work independently of the flow sensors and to adjust the PWM according to a set of time steps (Figure 5) which correlated with our reference flow profile (39, 40). To calibrate the pump, we first labeled the side of a water tub with a marker for the water volume at a level of 1 and 2 L. We then calculated the flow rate at which the pump was running by measuring the time that it took to fill a specific volume (e.g., 1 L) with water. The measured and desired flow rates were then compared and the linear correlation line was found and implemented into the code. For further verification, we took three trials at each designated constant flow rate (3–6 L/min) and plotted the adjusted flow rate and the desired flow rate (Figure 5A). We calibrated the Hall-Effect flow sensors by running water through a simulator loop and recording the number of spins the internal water turbine in each flow sensor underwent. We took this measurement at each flow rate recording every 10 s and calculated the average of 10 trials. Using the turbine spins and known flow rate, we then found a correction factor to convert spins per second into liters per minute. Finally, we verified that the flow sensor measurements accurately displayed on the LCD screen.

After verifying constant flow, we searched the literature to find accurate flow profiles for the inferior and superior vena cava for mimicking physiological pulsatile flow (Supplementary Table 1, Figure 5B) (39, 40). We converted the flow profiles into L/min and then applied fluid dynamics to a split tube system to calculate the required pump output (Figure 5B). In order to do this, we first found the cross-sectional area (*A*_*vc*_) of each tube in the system using equation (1):


(1) $$\begin{array}{l} A_{vc}=\pi \;\left({\frac{d_{v}}{4}}^{2}\right) \end{array}$$

Where *d*_*v*_is the respective diameter of the vessel. Once the area was found, we could calculate the velocity in each outgoing tube, which can be used to find the velocity of the inlet tube. Velocity (*v*_*v*_) is found from flow rate (*F*_*v*_) and cross-sectional area through the following equation:


(2) $$\begin{array}{l} v_{v}=\frac{F_{v}}{A_{v}} \end{array}$$

After calculating the velocities of the superior and inferior vena cava tubes, we could then find the input velocity (*v*_*IN*_) to the system–the velocity the pump would need to output–through a simple equation from fluid dynamics (41–43):


(3) $$v_{IN}=\frac{v_{SVC}{\left(d_{SVC}\right)}^{2}+\;v_{IVC}{\left(d_{IVC}\right)}^{2}}{{\left(d_{IN}\right)}^{2}}$$

Where IN represents the values for the inlet tube, SVC the superior vena cava tube, and IVC the inferior vena cava tube. The velocities change with time according to the variation in flow rate (Supplementary Table 1). One final conversion from velocity to flow rate was necessary to find the desired flow output of the pump. An algebraic adjustment to equation (2) creates the following equation for finding the flow rate from velocity and cross-sectional area:


(4) $$\begin{array}{l} F_{IN}=v_{IN}\;\times {\;A}_{IN} \end{array}$$

Upon calculating the flow rate the pump would need to output we implemented a timed stepping function to control the PWM and run the pump at physiological pulsatile flow. We verified pulsatile flow through calculating the theoretical time-weighted average flow over one cycle and measuring the average cycle flow over 10 cycles with the sensors. Meanwhile, we visually checked the leak at each connection in the flow line for 5–10 min during both constant and pulsatile flow settings.

To further understand the fluid behavior, we calculated wall shear stress (*τ*_*w*_) and wall shear rate (*γ*_*w*_) (43):


(5) $$\begin{array}{l} \tau_{w}=\frac{4\mu F}{\pi r^{3}} \end{array}$$

(6) $$\begin{array}{l} \gamma_{w}=\frac{4F}{\pi r^{3}} \end{array}$$

To do these theoretical calculations, we considered a Newtonian fluid–since we used water (μ = 1.0 cP) in our device–and found a value for each tube (SVC, IVC, and IN) at each time where *F* was the flow rate at that time and *r* was the radius of the tube (Supplementary Tables 2, 3) (32, 33, 44, 45).

### Catheterization Simulation

The simulator was filled with water and was set to the pulsatile flow pattern. A flow-directed catheter (swan-ganz catheter) provided by TZ Medical Inc. was manually inserted at the radial access site and the femoral access site, respectively. We visually checked whether the catheter could pass through the right chambers of the heart, through the pulmonary trunk, and into a pulmonary artery.

### Data Quantification

All the flow data is quantitatively analyzed. All the experiments were conducted at least three times. The quantitative data are presented as the mean ± standard deviation (SD) from more than at least three samples (*n* ≥ 3).

## Results and Discussion

### Design and Fabrication of the Heart and the Cardiovascular Flow System

One major challenge of our design was the anatomically accurate heart modeling. In order to verify that the heart model we had made was anatomically accurate, we used calipers to measure along the same axis as was measured on the cadaver heart casts (Table 2, Figure 3) (28, 34). Through averaging the small and large heart axes values found from the literature, the right atrial long axis ranges from 4.04 to 4.98 cm and the right atrial short-axis ranges from 4.32 to 5.26 cm. The right ventricular long axis ranges from 7.10 to 8.98 cm, the right ventricular short axis (maximal) ranges from 4.18 to 5.06 cm, and the right ventricular short-axis (midway) ranges from 3.28 to 4.16 cm. The values we measured for the various axes on our heart were 4.45, 5.08, 7.62, 5.00, and 4.81 cm, respectively, each of which fell within the range of the values provided by the literature (Table 2) (29–33). We didn't measure the areas because those were calculated through specific images of the silicone casts, which we didn't have the technology to perform. We are confident though that the anatomical accuracy is sufficient for initial testing of catheter function since the heart was reconstructed from MRI images and was validated through physical measurements. We also evaluated the length, size, and orientation of vessels (tubing). These were compared to blood vessel sizes from many references and are anatomically accurate (Table 3, Figure 1) (35–38).

Not only was it crucial to validate the anatomical accuracy of the heart, but we also needed to select a clear material with a similar coefficient of friction to a physiological heart. The cardiovascular system can have a coefficient of friction between 0.015 and 0.13 depending on the angle of the interacting surface (27, 46). Once we decided on a clear polyurethane with a D shore hardness of 80–which should theoretically give a coefficient of friction around 0.1–we set up a test to verify the frictional coefficient of the material. We measured the friction force for a sample puck of polyurethane by using a force sensor. By using the equation of frictional force ($\mu \;=\;\frac{Force}{Normal\;Force}$), we calculated the average coefficient of friction of the polyurethane to be 0.12 ± 0.05 which is within the range of physiological coefficients of friction. While it is within the range, our measured coefficient of friction is on the higher end which may cause the catheter to have more difficulty sliding along the wall of our manufactured heart than that of a human. However, it was the smoothest castable material available and did test within the range, therefore, the surface of the heart should have little enough friction for the needle of a catheter to move smoothly during catheter insertion.

### Fabrication of a Cardiovascular Flow Simulator

Once we built the most complicated part of the simulator, the polyurethane heart, we gathered the rest of the components (Figure 4) and assembled the entire design. An open/close ball valve was installed at both the inlet and outlet of the reservoir. Catheter insert ports were attached to the branch at the end of the femoral line and radial line *via* a Tuohy Borst adapter (Qosina Corp., USA). Both the femoral and radial access points had an 80 cm total tube length to the heart (Figures 2, 4). All the tubing was secured to a lightweight plastic pegboard backing with zip ties. The final dimensions of the peg board were 121.92 × 40.64 cm (length × width) with all other components of the device able to be oriented in a manner which fit the designated space. Finally, the pump, reservoir with the heating element and temperature sensor, and control box were all connected *via* a metal mounting frame. Overall, our device was easy to transport because of the lightweight plastic pegboard and mounting frame, was well secured (no parts fell off during transportation), and provided all the components necessary for a basic simulation of the cardiac venous system.

### Heating Element Test

The temperature of the system was controlled by a sous vide heating element designed to automatically moderate the temperature of the water and was monitored by a temperature sensor in the reservoir connected to the Arduino. We verified whether both the Arduino temperature sensor and heating element were working properly. To test, we filled a 3 L water tank and set the sous vide to heat it to 37°C. After heating for 5–10 m, we measured a water temperature of 36.56°C with the temperature sensor displayed on the LCD screen of the Arduino controller. Thus, we successfully verified the heating element and temperature sensor to control the fluid temperature of the simulator at 37°C.

### Leak Test

All the parts were properly assembled and tightened. We then investigated whether there was any leak in the loop including the heart model, connectors, and tubing under the condition of fluid circulation. In the first leak test, we observed leaks from the connectors with threading as the method of securing the link between the tubing and the connector. The threads in these connectors allowed for water to escape through the space between the connector and a tube because they were a continuous spiral between the water and the external air. To resolve this issue, we replaced the threading with a circular ridge structure to compress the tubing and create a tight seal without a pathway outside of the device (Figure 4). Eventually, with the new connectors, silicone sealant around the heart connections, and some hose clamps around all internal connectors (Figure 4), we observed no leaks from the simulator at any constant flow rate from 3 to 6 L/min or the pulsatile flow setting for over 5 min. Therefore, the system doesn't allow water to leak out of the connections or the heart.

### Flow Test

The system was designed to provide two types of flow patterns for creating hemodynamics. One was a continuous flow and the other was a pulsatile flow pattern to simulate typical venous flow at a resting heart rate and blood pressure. According to our design, we investigated flow rate and its pattern in the simulator. We tested varying rates of flow (3–6 L/min) controlled by the Arduino programmed code. Our plot of constant desired flow rate vs. output flow rate (Figure 5A) revealed a linear flow function. Ideally, the function between the desired and actual flow would be a relationship of y = x. Fitting a regression line to our verification data revealed a relationship of y = 1.012 × −0.0423, with an r^2^ = 0.9988, which has a difference that is acceptably small enough for our range of flow. Therefore, the continuous flow was validated from a range of 3–6 L/min using our calculated flow function.

Initially, to verify the pulsatile flow pattern, we planned on obtaining real-time flow values, however because the flow was varying on the order of milliseconds, the Arduino sampling rate limited our ability to obtain real-time data. Therefore, to verify the pulsatile flow, we calculated a time-weighted average flow over one cycle, which was 3.10 L/min (Table 4). We then synced the Arduino sensors to measure the flow every 10 cycles, which would give a reading of the desired average flow. Our measured average flow was 3.12 ± 0.13 L/min over three trials. Together, we successfully created a pulsatile flow pattern that replicates the physiologically relevant flow pattern in the cardiovascular system.

**Table 4:** The theoretical average flow rate vs. the measured average flow rate, standard deviation (SD), and standard error during pulsatile flow.

**Trials**	**Theoretical average flow rate (L/min)**	**Measured average flow rate (L/min)**
1	3.10	3.10
2	3.10	3.00
3	3.10	3.25
**Average ± SD**	3.10 ± 0.00	3.12 ± 0.13
**Standard error**	0.00	0.07

### Simulation of Catheterization

**Figure 6:**
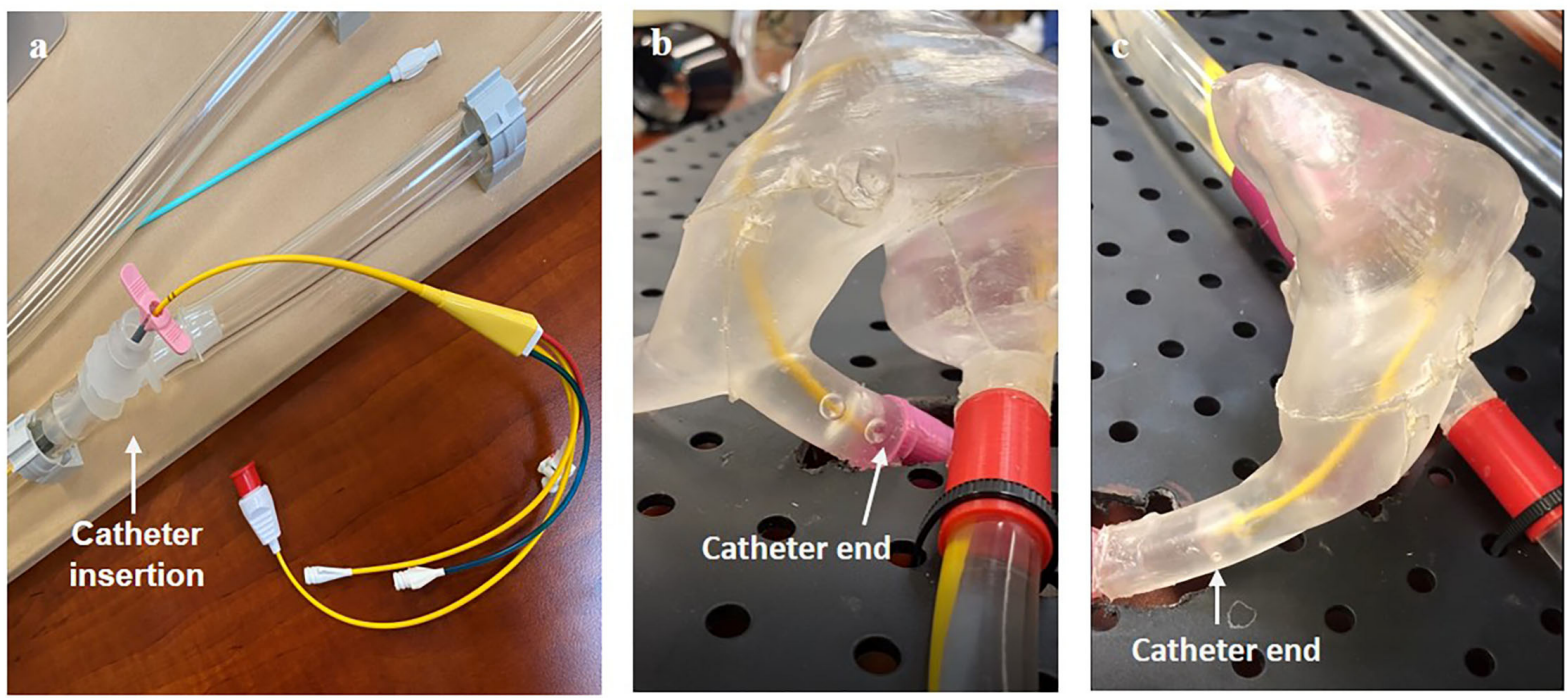
Visual verification of catheter function including **(a)** the method of insertion and the catheter through the right side of the heart **(b)** from a radial insertion point and **(c)** from a femoral insertion point.

To conduct a catheterization procedure simulation for femoral and radial access sites, we operated the system with the pulsatile flow pattern along with the operation of the sous vide heating element. Once the system stabilized at an average flow rate of 3.10 L/min and a temperature of 37°C, we inserted a flow-directed catheter into the system at a radial access site and visually observed the passage of the catheter through the complex anatomy of the vascular lines and heart (Figure 6, Supplementary Video 1). Next, we repeated the same process at a femoral access site and checked visually that the catheter passed through the heart to the pulmonary artery (Supplementary Video 2). Thus, we successfully verified the functional vascular flow simulator.

### Transportation

After using the simulator, the system can be turned off and the control box unplugged. The fluid is able to drain into the reservoir once the device is elevated and tilted above the return inlet. Compressed air can be used to flush out the fluid and dry it. Parts including the tubing, sensors, reservoir, and pump can be taken apart so that they are not difficult to replace if broken or worn out. The mounting frame for the control box, reservoir, and pump can also be disassembled for transportation purposes. Thus, our system is easy to transport, drain, and repair making it uncomplicated to maintain and relocate as necessary.

### Safety and Durability

To meet electrical safety regulations, the system was isolated from non-electrical components and all electrical components were installed and secured in the internal panel of the control box. To protect the system from shock, E-Stop, fuses, and a ground fault circuit interrupter (GFCI) breaker were installed and verified to shut off power upon activation. Next, we tested water resistance through a splash test and then performed a continuity test to ensure the fuses and GFCI protected the user and device. All the parts operated normally during the test. Additionally, the E-stop was verified by using it during normal operation of the vascular flow simulator and observing it stop all electrical activity. We also verified that the components worked long enough for the catheterization procedure through our catheterization simulation and observed no damage after the drop test. Thus, our simulator met the necessary safety regulations and provided a guarantee for no damage to critical components and safe operation of the system.

### Discussion of Results

Although we successfully constructed the cardiovascular flow simulator used for developing flow-directed catheter designs for venous insertion through either the femoral or radial access site (Figures 2, 5A), we need to improve our model to be more accurate anatomically and provide more realistic physiological functionality of the cardiovascular system before applying it to clinical training.

Perhaps the largest variance of our simulator design could be the absence of heart valves. In the initial design of our system, we did not include heart valves in order to focus on the development of the simulator for catheterization simulation. However, to replicate the more accurate heart model valves should be included despite the challenge of manufacturing and inserting them into the heart. We have a plan to include them in our model in the near future because we consider it important to also be able to replicate patients' diseased anatomical heart models (47). If our current model is modified by adding the heart valves to the system, it would not only be a more anatomical model, but could also be used for other purposes such as valve replacement simulation (48).

Another major limitation of our design is that the pump is separate from the heart model. Physiologically, the heart provides the source of fluid motion, however, practically implementing the heart as a pump is difficult because of its irregular shape. During our catheterization simulations, we noticed that pumping externally didn't incorporate the physiological fluid exertion from the apex of the heart which helps the catheter make the turn past the right ventricle into the pulmonary trunk. Some methods for fixing this issue in the future could be drilling a hole in the apex of the heart and attaching a separate tube to the pumping system so fluid enters the heart at the apex. Alternatively, a syringe could be attached to the hole and a manual pump could simulate the heartbeat.

In addition, if we want to consider using our system for patients' diseased heart models (48), it needs to provide various flow patterns to mimic irregular pulsatile flow of various heart diseases. Although our current system provides only two flow patterns–continuous and pulsatile flow patterns across the typical range of resting heart rates and blood pressures–it has a great potential to mimic flow patterns during heart diseases such as tachycardia or heart block. These flow patterns could easily be generated by modifying the Arduino programming code. An alternative way of producing other flow patterns could involve using a peristaltic pump instead of an impeller pump.

For further improvement of the system, two flow rate sensors at the inferior vena cava and the superior vena cava could be applied for verifying their individual flow patterns (Supplementary Table 1, Figure 5B). While one flow rate sensor verifies the general pulsatile flow pattern, adding two more would check for more physiological hemodynamics. Additionally, incorporating a microcontroller capable of a faster sampling rate would allow for real-time monitoring of the flow rate and further confirm the specific pulsatile pattern. It may even be helpful to use an entirely separate microcontroller for the flow sensors so the interrupts required to take samples quickly don't interfere with the pump control and other electronic components.

Next, it would be better if blood-like fluid was used in the system to create more physiological hemodynamics. In our study, we used water for testing the simulator to prove the concept and prototype function. Although blood behaves as a Newtonian fluid (apparent viscosity of about 3–4 cP) like water at high shear rates, since blood is a Casson fluid there are some behaviors which are different between blood and water (43). We expect that its effect might be negligible for catheterization simulation at the testing stage of the system. However, if blood-like fluid was desired, it is possible to use a mixture of glycerol and water at a ratio aligned with the viscosity of blood which would more precisely mimic physiological hemodynamics and may reveal some valuable information about catheter behavior (49, 50).

Overall, our system for catheterization procedure simulation allows clear visualization of catheter movement through vessels and chambers of the heart. It provides great benefits compared to the use of an ultrasound detector or x-ray to track medical device movement because it requires no operational skill for observation (51, 52). Economically, our approximate cost was $1,500 with the heart manufacturing process accounting for the highest percentage of that cost. Therefore, our system is cost-effective and relatively inexpensive for a cardiovascular simulator. It has the potential to grant smaller companies the opportunity to have a tool for doing some initial catheter design development without being overwhelmed by the cost of multiple, possibly avoidable, iterations of porcine model tests. We expect that our simulator would be used for medical device testing before using porcine models and could be used for proper catheter insertion training. Moreover, it is expected that medical companies might save on cost and time spent on animal testing before FDA approval of a medical device.

## Conclusions

We developed the anatomically and physiologically accurate cardiovascular flow simulator to help train medical professionals and to be used for testing medical devices. It replicates proper hemodynamics through pulsatile blood flow or constant flow from 3 to 6 L/min at body temperature (37°C) with an anatomically accurate adult heart and femoral and radial vessel access ports. We successfully demonstrated catheter insertion simulation by inserting a catheter into the system at radial/femoral access sites, passing it through the vascular line, and advancing it to the heart. Our model, operated by Arduino programming code, is cost effective, electrically safe, and transportable based on a modular platform for easy operation. Further, the simulator provides benefits to visualize the catheter movement and device's functionality. Thus, we expect that our simulator can be used as an educational tool in cardiac catheterization as well as a design/testing tool that will allow companies building catheters to iterate on their design before moving on to the animal testing stage.

## Data Availability Statement

The original contributions presented in the study are included in the article/Supplementary Material, further inquiries can be directed to the corresponding author.

## Author Contributions

AJ, GC, NA, DL, CS, and KW designed experiments, fabricated the devices, and performed the functional test of the system. AJ performed experiments regarding heart modeling, and device characterization. GC contributed to testing flow sensor, heating element, and Arduino program code. NA was in charge of fabrication of circuit. DL and CS conducted connector fabrication. KW contributed to 3D printing and simulation. YK, JM, CH, and NG analyzed and reviewed the data. AJ and YK wrote the manuscript. All authors discussed and interpreted the results, and edited the manuscript.

## Funding

This project was funded by TZ Medical Inc. as well as the KEEN grant for Entrepreneurially Minded Making (EMM) at George Fox University.

## Conflict of Interest

JM and CH are employed by TZ Medical Inc. This study received funding from TZ Medical Inc. The funders were involved in study design, data analysis, and decision to publish. The remaining authors declare that the research was conducted in the absence of any commercial or financial relationships that could be construed as a potential conflict of interest.

## Publisher's Note

All claims expressed in this article are solely those of the authors and do not necessarily represent those of their affiliated organizations, or those of the publisher, the editors and the reviewers. Any product that may be evaluated in this article, or claim that may be made by its manufacturer, is not guaranteed or endorsed by the publisher.
