# Supplementary material for: An Inexpensive Cardiovascular Flow Simulator for Cardiac Catheterization Procedure Using a Pulmonary Artery Catheter
Source: Front Med Technol. 2021 Oct 28;3:764007. doi: 10.3389/fmedt.2021.764007 (PMC8757711; doi:10.3389/fmedt.2021.764007)
Supplement: Supplementary file 1 [file Data_Sheet_1.docx]

**SUPPORTING INFORMATION**

**An inexpensive cardiovascular flow simulator for cardiac catheterization procedure using a pulmonary artery catheter**

*Annika Johnson*^1^*, Grace Cupp*^1^*, Nicholas Armour*^1^*, Kyle Warren*^1^*, Christopher Stone*^1^*, Davin Lee*^1^*, Nicholas Gilbert*^1^*, Chris Hammond*^2^*, John Moore*^2^*, and Youngbok (Abraham) Kang*^1*^

^1^ George Fox University, 414 N. Meridian St., #6088, Newberg, OR 97132 USA.

^2^ TZ Medical Inc, 17750 SW Upper Boones Ferry Rd #150, Portland, OR 97224 USA.

^*^Corresponding author: E-mail: ykang@georgefox.edu

**Supplementary heart reconstruction detailed explanation**

Since the MRI images from the AMRG atlas were stored in a dicom image format, an image software was required to view them. We used MATLAB with the image processing toolbox to import and view all of the images (Figure S1). The images were organized in various sets of different types (e.g., some showed blood flow others successive cross-sections) and labelled with increasing numbers upon download which made the processing simpler. Using the montage command in MATLAB, each image set was organized to display on an individual figure screen and visually replicate a video with each frame defined by the respective image in the set.


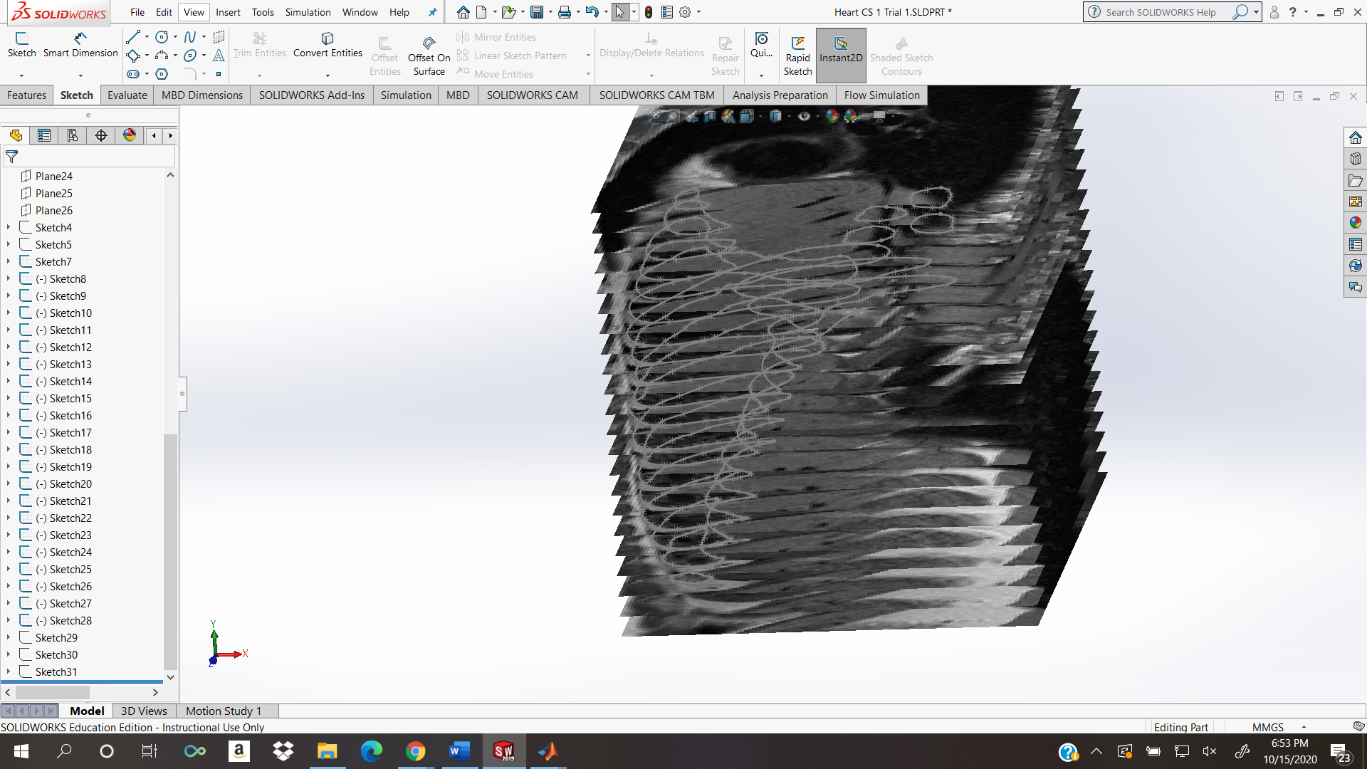


Figure S1. Individual plane sketches in SolidWorks of the right-side of the heart for reconstruction.

**Supplementary tables**

Table S1. Physiological flow rates for the superior vena cava (SVC) and inferior vena cava (IVC) over one cardiac cycle. We used these flow rates found from the literature to calculate the desired flow rate for the pump considering that it was feeding both ends.

| **Time (s)** | **SVC Flow (L/min)** | **IVC Flow (L/min)** | **Pump Flow (L/min)** |
| --- | --- | --- | --- |
| 0.00 | 0.00 | 0.00 | 0.00 |
| 0.12 | 3.04 | 3.67 | 6.71 |
| 0.24 | 1.52 | 3.12 | 4.64 |
| 0.40 | 0.76 | 2.76 | 3.52 |
| 0.42 | 1.67 | 2.88 | 4.55 |
| 0.54 | 2.74 | 3.00 | 5.74 |
| 0.70 | 0.76 | 1.63 | 2.39 |
| 1.00 | 0.00 | 0.00 | 0.00 |

Table S2. Calculated theoretical wall shear stress and shear rate for the SVC, IVC, and IN (tube inlet and pump outlet) for our device using water (a Newtonian fluid with $\mu=1.0 cP$) over a full cardiac cycle.

| **Time (s)** | **SVC Shear Stress (Pa)** | **IVC Shear Stress (Pa)** | **IN Shear Stress (Pa)** | **SVC Shear Rate (1/s)** | **IVC Shear Rate (1/s)** | **IN Shear Rate (1/s)** |
| --- | --- | --- | --- | --- | --- | --- |
| 0.00 | 0.000 | 0.000 | 0.000 | 0.000 | 0.000 | 0.000 |
| 0.12 | 0.252 | 0.078 | 0.142 | 252 | 77.9 | 142 |
| 0.24 | 0.126 | 0.066 | 0.098 | 126 | 66.2 | 98.5 |
| 0.40 | 0.063 | 0.059 | 0.075 | 63.0 | 58.6 | 74.7 |
| 0.42 | 0.138 | 0.061 | 0.097 | 138 | 61.1 | 96.6 |
| 0.54 | 0.227 | 0.064 | 0.122 | 227 | 63.7 | 122 |
| 0.70 | 0.063 | 0.035 | 0.051 | 63.0 | 34.6 | 50.7 |
| 1.00 | 0.000 | 0.000 | 0.000 | 0.00 | 0.00 | 0.00 |

Table S3. Comparison of our calculated wall shear stress and the wall shear stress from the literature for the SVC and IVC. The values of our wall shear stress are much smaller than those from the literature due to the difference in the viscosity, flow rate, and diameter for the SVC & IVC. Specifically, the water viscosity (1 cP) and flow rates (SVC: 0-3.04 L/min and IVC: 0-3.67 L/min) in our system are smaller than the blood viscosity (4-5 cP) and reference flow rates (SVC: 3.90-7.20 L/min and IVC: 2.40-6.36 L/min) (1). The diameter of the SVC (1.27 cm) in our system is relatively similar to or a little greater than the diameter of 1.41$\pm0.27$ cm in the literature (1, 2), but the diameter of the IVC (2.00 cm) in our system is greater than the diameter of 1.54$\pm$0.73 cm in the literature (1, 3). Although our system does not accurately replicate hemodynamics due to the large variation of human physiological data, we believe that our system still operates within the wide range of the physiological function of human hemodynamics.

|  | SVC in our system | SVC in the literature | IVC in our system | IVC in the literature |
| --- | --- | --- | --- | --- |
| Max. wall shear stress [Pa] | 0.25 | 2.40 (1) | 0.08 | 2.60 (1, 4) |
| Min. wall shear stress [Pa] | 0 | 1.40 (1) | 0 | 1.40 (1, 4) |
| Time-average wall shear stress [Pa] | 0.11 | 1.89 (1) | 0.05 | 1.89 (1) |

**Supplementary video files**

[Video S1. Catheterization Radial Catheter Test.MOV](https://www.youtube.com/watch?v=JsrXgoDlolI)

[Video S2. Catheterization Femoral Catheter Test.MOV](https://www.youtube.com/watch?v=lTx0YR0yWR4)

**Supplementary Arduino code for a temperature sensor, a flow sensor, and a motor**

//This code is designed to read and display temperature using the

//DS18B20 Compatible Digital temperature sensor and

//to read and display flow rate using the

//Water Flow Sensor YF-B2 sensors.

//It will also control the DC motor based on the flow rate input.

//Last Edited on 4/26/2021 by Grace Cupp

//Senior Design Vascular Flow Simulator 2020-2021

//============================================================

// Defined Variables and Libraries

//============================================================

//For Toggle Switches for Display and Type of Flow____________________________________________________________

//Display Switch

//Create a toggle variable to allow the switch function to be used

int togglePin = 8; // Assign the toggle switch to pin 8

int previous = LOW; // the previous reading from the toggle pin

long t = 0; // the last time the output pin was toggled

long debounce = 200; // the debounce time, increase if the output flickers

int toggleState = LOW; // the state of the toggle switch, HIGH or LOW that determines output

//Type of Flow switch

//Create another toggle variable to allow the system to switch between constant and pulsatile flow

int togglePin2 = 13; // Assign the toggle switch to pin 13

int previous2 = LOW; // the previous reading from the toggle pin

int toggleState2 = LOW;// the state of the toggle switch, HIGH or LOW that determines output

//________________________________________________________________________________________________________________

//For Temperature Sensor and LCD_______________________________________________________________________________

//Inlcude the required libraries to use the temperature sensor and LCD

#include <OneWire.h> // For the temperature sensor

#include <DallasTemperature.h> // For the temperature sensor

#include <LiquidCrystal.h> // For the LCD

//Define the bus that will be used with digital pin 5

#define ONE_WIRE_BUS 5 // Bus for the temperature sensor

OneWire oneWire(ONE_WIRE_BUS); // Assign the bus

DallasTemperature sensors(&oneWire);// Recognize the sensor

//Create variables to store the temperature in Fahrenheit and Celcius

float Celcius = 0;

float Fahrenheit = 0;

//Initialize the library by associating any needed LCD interface pins

//with the arduino pin number it is connected to

const int rs = 12, en = 11, d4 = 10, d5 = 4, d6 = 9, d7 = 7;

LiquidCrystal lcd(rs, en, d4, d5, d6, d7);

int stopClear; // Variable to stop the LCD from constantly clearing the display

//________________________________________________________________________________________________________________

//For Flow Meters_______________________________________________________________________________________________

volatile int flow_frequency1; // Measures flow sensor pulses unsigned to flow meter 1

volatile int flow_frequency2; // Measures flow sensor pulses unsigned to flow meter 2

float l_min1; // Calculated liters/min for flow meter 1

float l_min2; // Calculated liters/min for flow meter 1

unsigned char flowsensor1 = 3; // Sensor Input 1

unsigned char flowsensor2 = 2; // Sensor Input 1

unsigned long currentTime; // Current time in milliseconds

unsigned long cloopTime; // Current total loop time

//The hall-effect flow sensor

float calibrationFactor1 = 1; // Calibration factor for flow sensor 1

float calibrationFactor2 = 1; // Calibration factor for flow sensor 2

float sensorCoefficient1 = 19.5; // Flow sensor 1 calibration equation coefficient

float sensorIntercept1 = -12; // Flow sensor 1 calibration intercept

float sensorCoefficient2 = 19.5; // Flow sensor 2 calibration equation coefficient

float sensorIntercept2 = -12; // Flow sensor 2 calibration intercept

//________________________________________________________________________________________________________________

//For Motor_______________________________________________________________________________________________________

//Create variables to use in the pump equation so that input value equals output flowrate

float pumpSlope = 0.525; // Slope of pump equation

float pumpIntercept = 1.75; // Intercept of pump equation

int motorPin = 6; // Pin to control the DC motor

float flowProfileSVC[] = {40.0, 20.0, 10.0, 22.0, 35.0, 10.0, 0}; // Flow profile values for velocity in cm/s for SVC

float flowProfileIVC[] = {19.5, 16.5, 14.6, 15.3, 15.9, 8.66, 0}; // Flow profile values for velocity in cm/s for IVC

float DSVC = 1.27; // Diameter of SVC in cm

float DIVC = 2.00; // Diameter of the IVC in cm

float Din = 2.00; // Diameter of the input in cm

float minV = 0; // Minimum voltage of motor range

float maxV = 12; // Maximum voltage of motor range

int outputCounts = 255; // PWM resolution

unsigned long profileTimer; // Timer for the flow profile

float minL = 0; // Minimum flow in L/min for pump

float maxL = 16; // Maximum flow in L/min for pump

float L2V; // L/min to volts

float V2C; // Volts to counts

float L2C; // L/min to counts

int motorCounts; // Counts sent to motor

int initiator = 0; // Ensures that the counter for the pump starts after the setup code has been run

float pumpVelocity[] = {0, 0, 0, 0, 0, 0, 0}; // Velocity

float constantFlowRate = 5; // Set variable for constant flow reading from the range of 3-6

//=============================================================

// Setup Code

//=============================================================

void setup(void)

{

Serial.begin(19200); // Set the Baud rate

sensors.begin(); // Initialize the temperature sensor

lcd.begin(16, 2); // set up the LCD's number of columns and rows

pinMode(togglePin, INPUT); // Set up the toggle switch 1 as an input (output changer)

pinMode(togglePin2, INPUT); // Set up the toggle switch 2 as an input (output changer)

//For flow sensors__________________________________________________________________________________

// For flow sensor 1

pinMode(flowsensor1, INPUT); // Set up the flow sensor 1 as an input (output changer)

digitalWrite(flowsensor1, HIGH); // Internal Pull-Up

// For flow sensor 2

pinMode(flowsensor2, INPUT); // Set up the flow sensor 2 as an input (output changer)

digitalWrite(flowsensor2, HIGH); // Internal Pull-Up

attachInterrupt(0, pulseCounter1, RISING); // Setup Interrupt for the first pulse counter

attachInterrupt(1, pulseCounter2, RISING); // Setup Interrupt for the second pulse counter

//__________________________________________________________________________________________________

//Timers setup for the flow profile_________________________________________________________________

cli(); // Stop interrupts

// Set timer1 interrupt at 50Hz for the 20ms

TCCR1A = 0; // Set entire TCCR1A register to 0

TCCR1B = 0; // Same for TCCR1B

TCNT1 = 0; // Initialize counter value to 0

// Set compare match register for 1hz increments

OCR1A = 313; //= (16*10^6) / (50*1024) - 1;// (must be <65536)

// Turn on CTC mode

TCCR1B |= (1 << WGM12);

// Set CS10 and CS12 bits for 1024 prescaler

TCCR1B |= (1 << CS12) | (1 << CS10);

// Enable timer compare interrupt

TIMSK1 |= (1 << OCIE1A);

//___________________________________________________________________________________________________

sei(); // Enable interrupts

currentTime = millis(); // Set the currentTime variable equal to the millis() function

cloopTime = currentTime; // Set the cloopTime variable equal to the currentTime variable

//For Motor__________________________________________________________________________

pinMode(motorPin, OUTPUT); //Set the motor as an output

//Calculate the liters per minute and convert to counts to send to the motor

L2V = (maxL-minL)/(maxV-minV); //Liters per minute to volts calculation

V2C = (maxV-minV)/outputCounts; //Volts to counts calculation

L2C = L2V*V2C; //Liters per minute to counts

//Create the for loop to be able to use the toggle switch for the motor

for(int i = 0; i <= 6; i++)

{

//Define the state to change between continuous and pulsatile flow as an input

toggleState2 = digitalRead(togglePin2);

//Create the statement to have the output be pulsatile flow

if(toggleState2 == HIGH)

{

//Define the pump output as the pulsatile equation

pumpVelocity[i] = (((flowProfileIVC[i]*sq(DIVC)+flowProfileSVC[i]*sq(DSVC))/(sq(Din)))*(PI*(sq(Din/2.0))))*(3.0/50.0);

pumpVelocity[i] = pumpSlope*pumpVelocity[i] + pumpIntercept; // Input the pump calibration equation

//Serial.println(pumpVelocity[i]); // Serial print the pump velocity

}

//Create the statement to have the output be continuous flow

else

{

pumpVelocity[i] = constantFlowRate; // Define the pump output as the constant flow variable

pumpVelocity[i] = pumpSlope*pumpVelocity[i] + pumpIntercept; // Input the pump calibration equation

//Serial.println(pumpVelocity[i]); // Serial print the pump velocity

}

}

}

//Function for timer 1 to run PWM with the motor__________________________________________________________________

ISR(TIMER1_COMPA_vect)

{

//Initialization if statement

if(initiator == 0)

{

motorCounts = (7)/L2C; // Calculate motor counts

analogWrite(motorPin, motorCounts); // Write the counts to the motor

delay(10000); // Wait for a second to run the rest of the code

profileTimer = millis(); // Set the timer equal to the current time

initiator = 1; // Use the initiator variable to tell the system that initialization has been run

}

//Code to run the motor with PWM______________________________________________________________________________

if(millis()-profileTimer < 120)

{

motorCounts = (pumpVelocity[0])/L2C; // Calculate motor counts

analogWrite(motorPin, motorCounts); // Write the counts to the motor

}

if(millis()-profileTimer >= 120 && millis()-profileTimer < 240)

{

motorCounts = (pumpVelocity[1])/L2C; // Calculate motor counts

analogWrite(motorPin, motorCounts); // Write the counts to the motor

}

if(millis()-profileTimer >= 240 && millis()-profileTimer < 400)

{

motorCounts = (pumpVelocity[2])/L2C; // Calculate motor counts

analogWrite(motorPin, motorCounts); // Write the counts to the motor

}

if(millis()-profileTimer >= 400 && millis()-profileTimer < 420)

{

motorCounts = (pumpVelocity[3])/L2C; // Calculate motor counts

analogWrite(motorPin, motorCounts); // Write the counts to the motor

}

if(millis()-profileTimer >= 420 && millis()-profileTimer < 540)

{

motorCounts = (pumpVelocity[4])/L2C; // Calculate motor counts

analogWrite(motorPin, motorCounts); // Write the counts to the motor

}

if(millis()-profileTimer >= 540 && millis()-profileTimer < 700)

{

motorCounts = (pumpVelocity[5])/L2C; // Calculate motor counts

analogWrite(motorPin, motorCounts); // Write the counts to the motor

}

if(millis()-profileTimer >= 700 && millis()-profileTimer < 1000)

{

motorCounts = (pumpVelocity[6])/L2C; // Calculate motor counts

analogWrite(motorPin, motorCounts); // Write the counts to the motor

}

if(millis()-profileTimer >= 1000)

{

profileTimer = millis(); // Reset the timer to the millis() function

}

}

//_____________________________________________________________________________________________________________

//==============================================================================

// Main Program Code

//==============================================================================

void loop(void)

{

//Code to run and switch between the output LCD display________________________________________________________________

//Define the state to change between temperature and flow rate display as an input

toggleState = digitalRead(togglePin);

//Create the if statement for the toggle switch to print on the LCD

if(toggleState == HIGH && previous == LOW && millis() - t > debounce && stopClear == 0)

{

lcd.clear(); // Clear the LCD screen of prevoius values

t = millis(); // Set the time for the last toggle to the millis() function

stopClear = 1; // Use the variable to stop the LCD from constantly clearing

}

//Create the statement to have the output be temperature reading

if(toggleState == HIGH)

{

lcd.setCursor(7, 0); // Set the cursor to column 7, line 0

lcd.print(" C "); //LCD print the letter C to distinguish Celcius reading

temperature(); // Run the temperature() function

}

//Create the statment to have the output be flow rate reading

else

{

lcd.setCursor(7, 0); // Set the cursor to column 7, line 0

lcd.print(" "); // Get rid of the letter C

lcd.setCursor(0, 0); // Set the cursor to column 0, line 0

lcd.print("Flow 1: ");// Let the user know what is printing

lcd.setCursor(0, 1); // Set the cursor to column 0, line 0

lcd.print("Flow 2: ");// Let the user know what is printing

flow(); // Run the flow() function

stopClear = 0; // Stop clearing the LCD so that it's not constant

}

//Define the state to change between continuous and pulsatile flow as an input

toggleState2 = digitalRead(togglePin2);

//Create the statement to have the output be pulsatile flow

if(toggleState2 != previous2)

{

previous2 = toggleState2; // Make the prevoius2 variable equal to the toggleState2 variable

//Create the for loop to be able to use the toggle switch for the motor

for(int j = 0; j <= 6; j++)

{

//Create the statements to change between continuous and pulsatile

//Create the statement to have the output be pulsatile flow

if(toggleState2 == HIGH)

{

//Define the pump output as the pulsatile equation

pumpVelocity[j] = (((flowProfileIVC[j]*sq(DIVC)+flowProfileSVC[j]*sq(DSVC))/(sq(Din)))*(PI*(sq(Din/2.0))))*(3.0/50.0);

pumpVelocity[j] = pumpSlope*pumpVelocity[j] + pumpIntercept; // Input the pump calibration equation

//Serial.println(pumpVelocity[i]); // Serial print the pump velocity

}

//Create the statement to have the output be continuous flow

else

{

pumpVelocity[j] = constantFlowRate; // Define the pump output as the constant flow variable

pumpVelocity[j] = pumpSlope*pumpVelocity[j] + pumpIntercept; // Input the pump calibration equation

//Serial.println(pumpVelocity[i]); // Serial print the pump velocity

}

}

}

}

//Create a function to print the temperature reading

void temperature()

{

//For Temperature Sensor___________________________________________________________________________________

sensors.requestTemperatures(); // Request the temperature to be read from the sensor

Celcius = sensors.getTempCByIndex(0); // Get the temperature value in Celcius

Fahrenheit = sensors.toFahrenheit(Celcius); // Convert the temperature to Fahrenheit

//________________________________________________________________________________________________________

lcd.setCursor(0, 0); // Set the cursor to column 0, line 0

lcd.print(Celcius); // LCD print the temperature in degrees Celcius

lcd.setCursor(0, 1); // Set the cursor to column 0, line 1

lcd.print(millis() / 1000); // Print the number of seconds since reset

}

//Create a function to print the flow reading

void flow()

{

currentTime = millis(); // Set the currentTime variable equal to the millis() function

//Every 10 seconds, calculate and print liters/min

if(currentTime >= (cloopTime + 10000))

{

cloopTime = currentTime; // Set the cloopTime variable equal to the currentTime variable

//Calibration equation for flow sensor 1 (connected to pin 2)

calibrationFactor1 = sensorCoefficient1*log(flow_frequency1)+sensorIntercept1;

//Output reading for flow sensor 1

l_min1 = (flow_frequency1 / calibrationFactor1);

//Serial.print("FF1 ");

Serial.print(flow_frequency1);

Serial.print("\t");

flow_frequency1 = 0; // Reset counter for flow sensor 1

//Serial.print(l_min1, DEC); // Print L/min

//Serial.println(" L/min");

//Serial.print("\t\t");

//Calibration equation for flow sensor 2 (connected to pin 3)

calibrationFactor2 = sensorCoefficient2*log(flow_frequency2)+sensorIntercept2;

//Output reading for flow sensor 2

l_min2 = (flow_frequency2 / calibrationFactor2);

//Serial.print("FF2 ");

Serial.println(flow_frequency2);

//Serial.print("\t\t\t");

flow_frequency2 = 0; // Reset counter for flow sensor 2

//Serial.print(l_min2, DEC); // Print L/min

//Serial.println(" L/min");

}

lcd.setCursor(10, 0); // Set the cursor to column 0, line 0

lcd.print(l_min1); // Print the integer part of the variable EDITED from int(flowRate1)

lcd.setCursor(10 ,1); // Set the cursor to column 0, line 0

lcd.print(l_min2); // Print the integer part of the variable EDITED from int(flowRate1)

}

//Create functions to count the pulses for the flow meters

/*

Interrupt Service Routine

*/

void pulseCounter1()

{

flow_frequency1++; // Increment the pulse counter

}

void pulseCounter2()

{

flow_frequency2++; // Increment the pulse counter

}

Reference

1. de Oliveira DC, Owen DG, Qian S, Green NC, Espino DM, Shepherd DET. Computational fluid dynamics of the right atrium: Assessment of modelling criteria for the evaluation of dialysis catheters. PloS one. 2021;16(2):e0247438.

2. Tang E, Restrepo M, Haggerty CM, Mirabella L, Bethel J, Whitehead KK, et al. Geometric characterization of patient-specific total cavopulmonary connections and its relationship to hemodynamics. JACC Cardiovascular imaging. 2014;7(3):215-24.

3. Weekes AJ, Tassone HM, Babcock A, Quirke DP, Norton HJ, Jayarama K, et al. Comparison of serial qualitative and quantitative assessments of caval index and left ventricular systolic function during early fluid resuscitation of hypotensive emergency department patients. Academic emergency medicine : official journal of the Society for Academic Emergency Medicine. 2011;18(9):912-21.

4. Cheng CP, Herfkens RJ, Taylor CA. Inferior vena caval hemodynamics quantified in vivo at rest and during cycling exercise using magnetic resonance imaging. American journal of physiology Heart and circulatory physiology. 2003;284(4):H1161-7.
